# *Euphorbia*-Derived Natural Products with Potential for Use in Health Maintenance

**DOI:** 10.3390/biom9080337

**Published:** 2019-08-02

**Authors:** Bahare Salehi, Marcello Iriti, Sara Vitalini, Hubert Antolak, Ewelina Pawlikowska, Dorota Kręgiel, Javad Sharifi-Rad, Sunday I. Oyeleye, Adedayo O. Ademiluyi, Katarzyna Czopek, Mariola Staniak, Luísa Custódio, Ericsson Coy-Barrera, Antonio Segura-Carretero, María de la Luz Cádiz-Gurrea, Raffaele Capasso, William C. Cho, Ana M. L. Seca

**Affiliations:** 1 Student Research Committee, School of Medicine, Bam University of Medical Sciences, Bam 44340847, Iran; 2 Department of Agricultural and Environmental Sciences, Milan State University, via G. Celoria 2, 20133 Milan, Italy; 3 Institute of Fermentation Technology and Microbiology, Lodz University of Technology, Wolczanska 171/173, 90-924 Lodz, Poland; 4 Zabol Medicinal Plants Research Center, Zabol University of Medical Sciences, Zabol 61615-585, Iran; 5 Functional Foods and Nutraceuticals Unit, Department of Biochemistry, Federal University of Technology, Akure 340252, Nigeria; 6 Department of Biomedical Technology, Federal University of Technology, Akure 340252, Nigeria; 7 Institute of Soil Science and Plant Cultivation – State Research Institute, Czartoryskich 8, 24-100 Puławy, Poland; 8 Centre of Marine Sciences, University of Algarve, Faculty of Sciences and Technology, Building 7, Campus of Gambelas, 8005-139 Faro, Portugal; 9 Bioorganic Chemistry Laboratory, Facultad de Ciencias Básicas y Aplicadas, Universidad Militar Nueva Granada, Campus Nueva Granada, Cajicá 250247, Colombia; 10 Department of Analytical Chemistry, Faculty of Sciences, University of Granada, Avda. Fuentenueva s/n, 18071 Granada, Spain; 11 Research and Development Functional Food Centre (CIDAF), Bioregión Building, Health Science Technological Park, Avenida del Conocimiento s/n, 188016 Granada, Spain; 12 Department of Agricultural Sciences, University of Naples Federico II, 80055 Portici, Italy; 13 Department of Clinical Oncology, Queen Elizabeth Hospital, Hong Kong SAR, China; 14 cE3c- Centre for Ecology, Evolution and Environmental Changes/ Azorean Biodiversity Group & University of Azores, Rua Mãe de Deus, 9501-801 Ponta Delgada, Portugal; 15 QOPNA & LAQV-REQUIMTE, Department of Chemistry, University of Aveiro, 3810-193 Aveiro, Portugal

**Keywords:** *Euphorbia*, essential oils, extracts, phytochemicals, terpenoids, bioactivity, antimicrobial, anti-inflammation, anticancer

## Abstract

*Euphorbia* genus (Euphorbiaceae family), which is the third largest genus of angiosperm plants comprising ca. 2000 recognized species, is used all over the world in traditional medicine, especially in the traditional Chinese medicine. Members of this taxa are promptly recognizable by their specialized inflorescences and latex. In this review, an overview of *Euphorbia*-derived natural products such as essential oils, extracts, and pure compounds, active in a broad range of biological activities, and with potential usages in health maintenance, is described. The chemical composition of essential oils from *Euphorbia* species revealed the presence of more than 80 phytochemicals, mainly oxygenated sesquiterpenes and sesquiterpenes hydrocarbons, while *Euphorbia* extracts contain secondary metabolites such as sesquiterpenes, diterpenes, sterols, flavonoids, and other polyphenols. The extracts and secondary metabolites from *Euphorbia* plants may act as active principles of medicines for the treatment of many human ailments, mainly inflammation, cancer, and microbial infections. Besides, *Euphorbia*-derived products have great potential as a source of bioactive extracts and pure compounds, which can be used to promote longevity with more health.

## 1. Introduction

The genus *Euphorbia* (Euphorbiaceae) is the third major genus of flowering plants, with 1836 accepted species [1,2], subdivided into many subgenera and sections. This genus has a worldwide distribution and can be found in all temperate and tropical regions. Also, this group of plants is characterized by an extraordinary variety of forms, from small ephemerals to several forms of herbaceous annuals or perennials, big shrubs, small trees, cushion-forming subshrubs, and cactus-like succulents [3]. From the 243 *Euphorbia* species assessed by the IUCN Red List of Threatened species, 170 (70%) are threatened with extinction (categories vulnerable, endangered, and critically endangered) [4].

More than 5% of species of *Euphorbia* are used in traditional medicine, mainly as emetic and purgative agents, to treat digestive and respiratory disorders, skin and inflammatory conditions, migraine, intestinal parasites and gonorrhoea, and as wart cures [5,6,7,8,9]. The usable parts of the *Euphorbia* species include roots, seeds, latex, wood, barks, leaves, and whole plants [5,6,7,8,9]. A brief overview of traditional medicine applications of *Euphorbia* is described in Section 2.

*Euphorbia* species have these curative properties due to the presence of various phytochemicals, which constitute the secondary metabolites of these plants [1,10,11,12,13,14,15,16,17]. They belong mainly to the terpenoids, flavonoids and polyphenols classes which also exhibit a great variety of biological effects such as cytotoxic, mammalian mitochondrial respiratory chain inhibition, HIV-1 and bacterial infection inhibition, anti-inflammatory, multidrug resistance modulators [13,18,19,20,21,22,23]. In fact, there is a good attention in *Euphorbia*-derived metabolites mainly because of the diterpene ingenol mebutate identified on *E. peplus* L. (as well as on *E. lathyris* L., *E. nivulia* Buch.-Ham., *E. esula* L., *E. antiquorum* L., *E. serpens* Kunth, and *E. fischeriana* Steud.), and is the active ingredient of Picato® medicine used in topical therapy against the precancerous skin condition actinic keratosis [24,25,26]. However, some *Euphorbia* compounds are toxic, resulting from an evolutionary strategy of plant defence against predators (e.g., herbivores), compounds that have a caustic and irritating effect to the skin and promote tumours [10,27].

*Euphorbia* plants are easily distinguishable by their toxic and highly skin irritant milky latex and particular inflorescences, designated as cyathia [28,29], and are widely used as ornamental plants, such as *E. milii* Des Moul., *E. tirucalli* L., and *E. lactea* Roxb [30]. The latex is the most valuable product obtained from *Euphorbia* species despite being toxic, it contains several biologically active natural compounds, such as triterpenoids [31]. Besides, latex is used in commercially valuable products like paints and natural rubber (intisy rubber obtained from *E. intisy* Drake) [30,32].

Secondary metabolites contained in *Euphorbia* plants also potentiate their use for food preservation. According to Toro-Vazquez et al. [33], candelilla wax obtained from the leaves of some species of *Euphorbia* found in Northern Mexico and the Southwest of the United States was recognized by the Food and Drug Administration (FDA) as a food additive with gelling properties, forming oleo-gels together with vegetable oils. According to EU regulations, candelilla wax is assigned by E902 additive code, and it is also an allowed glazing agent, applied on the surface of confectionery, nuts, wafers, coffee grains, dietary supplements, and fresh fruit [34].

Taking into account the great interest of the *Euphorbia* plants, we aim to touch on the chemical composition of essential oils, the therapeutic potential, in vitro, in vivo, and clinical trials of *Euphorbia* extracts and the pure compounds. We adopt the Latin binomial taxonomic name of the *Euphorbia* species considered by the Plant List database. When it does not match with the taxonomic name indicated in the bibliographic reference, the synonym will be shown in parentheses.

## 2. Traditional Medicine Uses of *Euphorbia* Plants

The *Euphorbia* genus is well-known to involve several plants used in folk medicine in different parts of the world, especially in traditional Chinese medicine [5,7,9]. Moreover, a recent study discriminated the global geographical distribution regarding uses of *Euphorbia* plants in traditional medicine [6]. In this regard, three particular uses were most often detected, such as (1) treatments of digestive system disorders (very globally frequent excepting Australasia); (2) as remedies for infections/infestations (mainly in Southern Africa and America, Pacific, Asia-tropical, and Asia-temperate); and (3) for treating skin/subcutaneous cellular tissue disorders (particularly in Australasia, Europe, Asia, and Northern America). On the other hand, within the 33 species with citations in folk practices worldwide, the three most-referenced plants used as traditional medicines were *E. hirta* L., *E. thymifolia* L., and *E. lathyris* [6].

*Euphorbia hirta* whole plant has been employed in Burundi, China, Philippines, and Nigeria to manage diarrhoea [35,36,37,38], while *E. hirta* decoction is used in Vietnam, India, and Mozambique to treat dysentery [39,40,41] and to treat bronchitis/asthma/coughs in Nepal, Australia, the South Western United States, and Hawaii [6,39,42]. Additionally, the latex from *E. hirta* is also applied to treat skin diseases and fever mostly in Asia [6] and to treat gonorrhoea in Malaysia [43] and other conditions such as malaria, candidiasis, and ringworm infections [6]. Populations aroundthe Vellore District of Tamil Nadu, India, use decoction of the *E. hirta* whole plant to treat poisonous snakebites (topically and orally administration) [44].

Despite the registered abortifacient properties of *E. thymifolia* decoctions in Chile, its latex or leaf decoctions have been recorded as lactation stimulants in different continents [45]. In the case of *E. lathyris*, emetic and purgative actions have been described in Europe as well as its seeds used to treat snakebites, ascites, schistosomiasis, and hydropsy [38,46].

*Euphorbia maculata* L. in Northern America is used for the treatment of corneal opacities and warts [47], while in China, it is used to treat blood disorders (e.g., haematuria, haemoptysis, epistaxis, and hemafecia), carbuncles, and wounds [38]. *Euphorbia denticulata* Lam. and *E. macrocarpa* Boiss. & Buhse are also used for wound healing in Turkey [48], and a similar use is reported in Ethiopia for *E. heterophylla* L. and *E. prostrata* Aiton [49].

The decoction, unguent, or hot steam of other *Euphorbia* species are used on inflammation conditions, such as *E. corollata* L. (for dropsy), *E. marginata* Pursh, and *E. antiquorum* (for swellings) [6]. Similarly, *E. antiquorum* is utilized in Vietnam to alleviate toothache events [41] as well as for treating cutaneous dropsy, cutaneous infections, cancer, and liver ailment [50]. *E. tirucalli* L. and *E. ingens* E.Mey. ex Boiss. like *E. lathyris*, can be used as an emetic against snakebites [39,51]. A recent review has been published showing that *E. tirucalli* (whole plant and its parts individually separated) has some records in South America, India, the Middle East, and Africa regarding beneficial effects on leprosy, syphilis, cancer, asthma, and intestinal parasites [51]. The same research group [52] also published a review where they report the various applications in traditional medicine of *E. neriifolia* L. Its latex is used as a carminative and expectorant, as well as in the treatment of tumours, abdominal and skin problems, leprosy, asthma, and kidney stones, while the roots are used in the treatment of scorpion stings and snake bites. The leaves can also be used as carminative and in the treatment of pain, inflammation, bronchial infections and lack of appetite [52]. *Euphorbia helioscopia* L. is used in the traditional Chinese medicine in situations of bacillary dysentery, osteomyelitis, and malaria [53]. In Uyghur medicine, China, *E. resinifera* O.Berg is recurrently employed to suppress tuberculosis, toothache, and chronic pain [54], while *E. fischeriana* have been used as a remedy for cancer, ascites, and oedema [55], and *E. granulata* Forssk. is utilized against intestine worms, oedema, cough, blood impurities, and renal diseases [56,57].

However, some *Euphorbia* plants, especially their latex or milky sap (e.g., *E. hirta*, *E. helioscopia, E. royleana* Boiss. among others), are considered as irritating materials for skin, mouth, and throat, causing burning sensation, acute inflammation (even blisters), and nausea [58]. In veterinary medicine, *E. milii* Des Moul. and *E. nivulia* is used to treat diarrhoea and wounds in livestock, respectively, but other *Euphorbia* species can produce irritations [6].

## 3. *Euphorbia* Plants: Essential Oil Composition and Activities

**Figure 1:**
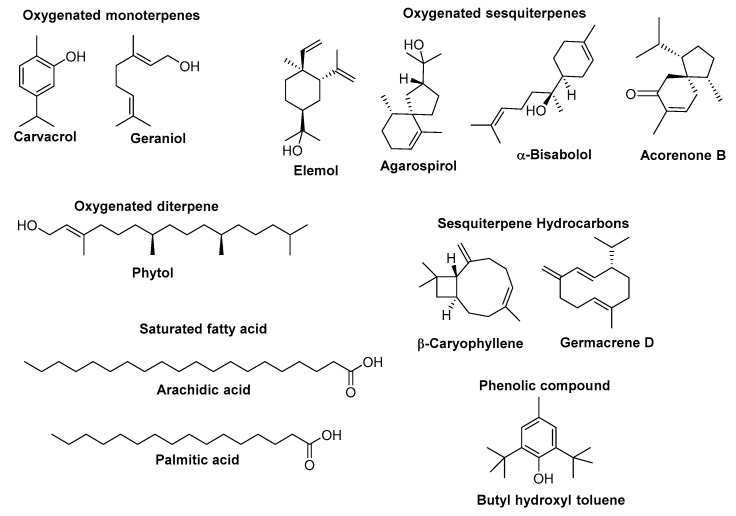
Chemical structures of the constituents of *Euphorbia* essential oil, each one with a content exceeding 25%.

Researchers from various countries worldwide have studied the chemical composition of essential oils (EOs) from different *Euphorbia* species. An overview of their most abundant components (the content higher than 5%) along with the most relevant biological activities to health maintenance (when available, and when the biological activity of a positive standard compound was also presented) is given in Table 1. The chemical structure of the major constituents of EOs from *Euphorbia* species whose content is higher than 25% is depicted in Figure 1.

The Table 1 data show that EOs were obtained mainly from aerial parts (39%) and inflorescences (29%), in addition to leaves (18%), roots (11%), and stems (3%), by using basically two extraction methods—hydro-distillation (HD) (52%) and steam distillation (SD) (45%). The oil yield ranged from 0.07% to 1.52% (*w*/*v*) in *E. cotinifolia* (syn. *E. caracasana*) and *E. fischeriana*, and from 0.08% to 0.84% (*w*/*w*) in *E. pilosa* and *E. densa*. Microwave-assisted extraction (MAE) was reported only once (3%) with faster extraction time (3:1) and higher oil yield (1.2% vs. 0.7% *w*/*v*) than conventional techniques (MAE vs. HD) [74]. Qualitative and quantitative analyses were performed by gas chromatography (GC) or GC coupled to mass spectrometry (GC-MS). Samples were found to contain from 8 to 83 phytochemicals representing 81.7–99.9% of the oils content. Oxygenated sesquiterpenes (up to 86.1% of the oil in *E. teheranica*) characterize EOs of *Euphorbia* species, followed by sesquiterpene hydrocarbons (up to 34.8% in *E. helioscopia*) (Table 1). In general, *β*-caryophyllene was the most ubiquitous sesquiterpene present in 50% of the species investigated namely in *E. acanthothamnos*, *E. apios*, *E. cotinifolia, E. densa, E. fischeriana, E. fragifera*, *E. golondrina*, *E. helioscopia, E. heterophylla, E. rigida*, *E. sanctae-caterinae*, *E. teheranica* and *E. tithymaloides* constituting more than 7% of their EOs (Table 1; Figure 1).

As reported by Lokar et al. [63], different habitats can influence the quantitative composition of EO from the same species. For example, EO of *E. fragifera* growing in a xeric habitat was richer in aromatic terpenes than that obtained from plants collected in shady and moist soils (e.g., 61.55% vs. 3.36% of carvacrol) being the last ones characterized by great quantity of acyclic compounds (e.g., 1.24% vs. 59.65% of geraniol). Moreover, variation in the components of EOs may occur due to the season, geographical area, and date of collection [63].

From Table 1, it appears that most of the EOs of *Euphorbia* species studied exhibit antioxidant properties, especially by the radical scavenging mechanism. Note that some of them are more active than ascorbic acid, BHT, or BHA compounds well known for their antioxidant properties and are widely used in the food industry as a preservative.

On the other hand, the data presented also show that there are many *Euphorbia* species whose EOs are still not yet studied, thus evidencing a knowledge gap about the potential of these species.

## 4. Antibacterial and Antifungal Activity of *Euphorbia* Plants

Plants belonging to the genus *Euphorbia* are also of the great interest in the matter of their antimicrobial activity [51,52,78,79,80]. In fact, these plants are also widely used in the traditional medicine in the microbial infections [81,82], and some *Euphorbia* plants are believed to be a promising source of phytochemicals used in the pharmacy and food industries [83]. Since consumers prefer healthy products without synthetic raw materials, the constantly growing interest in the natural and ecologically friendly antimicrobial agents is still being observed, and therefore research on the antimicrobial activity involving *Euphorbia* species is relevant. The most recent studies in this subject and with greater impact is summarized in Table 2. This is not an exhaustive list of all studies performed, and only those that meet as a minimum requirement the indication of at least one statistical parameter associated with the average value of activity level (e.g., standard deviation) are included.

According to Ashraf et al. [84], the hexane extract of *E. royleana*, when compared with methanol and water extracts, is the one with highest phenolic and flavonoid contents and the best antimicrobial agent. In fact, it exhibits antifungal activity against *Aspergillus niger* and antibacterial activity against the gram-positive bacteria *Bacillus subtilis*, about half that of the reference compounds (rifampicin and terbinafine).

The methanol extracts of *E. hirta* L. and *E. tirucalli* L. exhibit similar activity against a broad spectrum of bacteria and fungi associated with skin infections (zones of growth inhibition ranged from 9.0 mm (*B. subtilis*) to 14.0 mm (*P. aeruginosa*)) [85]. These results support the use of these species in traditional Indian medicine, and they can be used as an easily accessible source of natural antimicrobial agents [85].

The results obtained by Kumara Swamy et al. [86] showed that, independent of the extract prepared and the microorganism tested, *E. neriifolia* exhibits low antibacterial and antifungal activity, being the less interesting *Euphorbia* species as a source of antimicrobial agents.

In another study, the chloroform extract of *E. paralias* L. stems seems to be the most interesting extract, since it exhibits similar activity against fungi, gram-positive, and gram-negative bacteria (MIC = 15 µg/mL against *C. albicans*, *B. subtilis*, and *E. coli* strains, Table 2), while the chloroform extract of leaves only exhibits identical activity against *C. albicans*. The acetone extracts exhibit low activity against all the microorganisms tested [87].

According to the study conducted by Awaad et al. [88], the ethanolic extract of *E. hirta* aerial parts was the most active against all the bacteria and fungi tested when compared with *E. granulata* and *E. helioscopia* ethanol extracts, exhibiting an antifungal activity against *M. canis* similar to the amphotericin B in the same experimental conditions. Moreover, the authors demonstrated that heptacosan-1-ol, isolated from the active extract, could be the main metabolite responsible to the antimicrobial activity of *E. hirta* extract.

The study conducted by Ogbulie et al. [89] revealed that ethanolic extracts of *E. hirta* leaves showed higher activity against the gram-positive bacteria *S. aureus* (MIC = 22.55 mg/mL) than against the other microorganisms tested, but it was much less active than the ethanol extract of aerial parts against the same *S. aureus* strain (MIC= 1.95 µg/mL).

Unlike the ethanolic extract from the leaves of *E. hirta*, that does not inhibit the growth of *Salmonella typhi*, the ethanolic extract of aerial part from this species used in the study of Perumal et al. [90] that exhibited the strongest antimicrobial activity against *Salmonella typhi* with MIC value of 31 µg/mL, an activity higher than the chloramphenicol activity against the same strain (Table 2). Additionally, the same extract (the ethanolic extract of aerial part) also exhibits, against the gram-negative bacteria *Proteus vulgaris*, higher activity (MIC = 250 µg/mL) than the reference compounds gentamicin and chloramphenicol (MIC = 500 µg/mL) [90]. On the other hand, hexane extract was not active against all the tested microorganisms, except a weak activity against *P. vulgaris* [90].

Pisano et al. [91] carried out relatively extensive studies on the antimicrobial activity of the aqueous and ethanolic extracts from leaves, stems, and flowers of *E. characias* L. (Table 2). The results showed that most of these extracts are non-active against the organisms tested (MIC > 1.25 mg/mL), with the exception of the ethanolic extract from leaves against gram-positive bacteria *Bacillus subtilis* (MIC = 312.5 µg/mL).

## 5. Other Biological Activity of *Euphorbia* Plants

The *Euphorbia* plants have been used as medicinal plants for the treatment of many human ailments besides to those caused by bacteria and fungi. In fact, as will be demonstrated in the items below, *Euphorbia* extract and compounds have been evaluated as potential natural drugs with many other activities such as anti-inflammatory, antiviral, and antitumour.

### 5.1. In Vitro Studies

Studies on in vitro biological activity of several *Euphorbia* plants have been reported, involving both extracts and pure compounds.

The cytotoxicity of the crude extract of the stem bark of *E. umbellata* (Pax) Bruyns and its fractions (hexane, chloroform, ethanol, and methanol) were studied using in vitro assay and by applying the leukemic cells Jurkat clone E6-1 [92]. The study revealed that the CHCl_3_ fraction had the highest cytotoxicity (IC_50_ = 7.72 µg/mL), an activity value much lower than the reference compound vincristine (IC_50_ = 0.0031 µg/mL), but below the limit 30 µg/mL. According to NCI criteria [93], the activity of extracts is considered interesting to be studied in more detail. The established mechanism responsible for this action was associated with the promotion of cell cycle arrest at G0/G1 phase and apoptosis, which was related to two main classes of compounds in this fraction—steroids and triterpenes [92].

More recently, ethanolic extract of latex from *E. umbellata* has been evaluated for its anti-HIV properties. The study demonstrated in several models (in resting CD_4_+T cells, in cells from HIV-1 + individuals, and in primary non-human primate CD4+T cells) that this extract is able to reactivate the HIV virus in latency, acting on three factors with synergistic effect [94].

The ethanolic extract of *E. lunulata* Bunge (this name is a synonym of *E. esula* L.), at 10–20 µg/mL, had a significant anti-proliferative, anti-migration, and anti-invasion effect on multidrug resistant human gastric SGC7901/ADR cells, being able to arrest cell cycle progression and to induce cell apoptosis [95]. The same study further revealed that the cell cycle was arrested in G_2_/M phase, while significant apoptotic morphological changes was observed.

**Figure 2:**
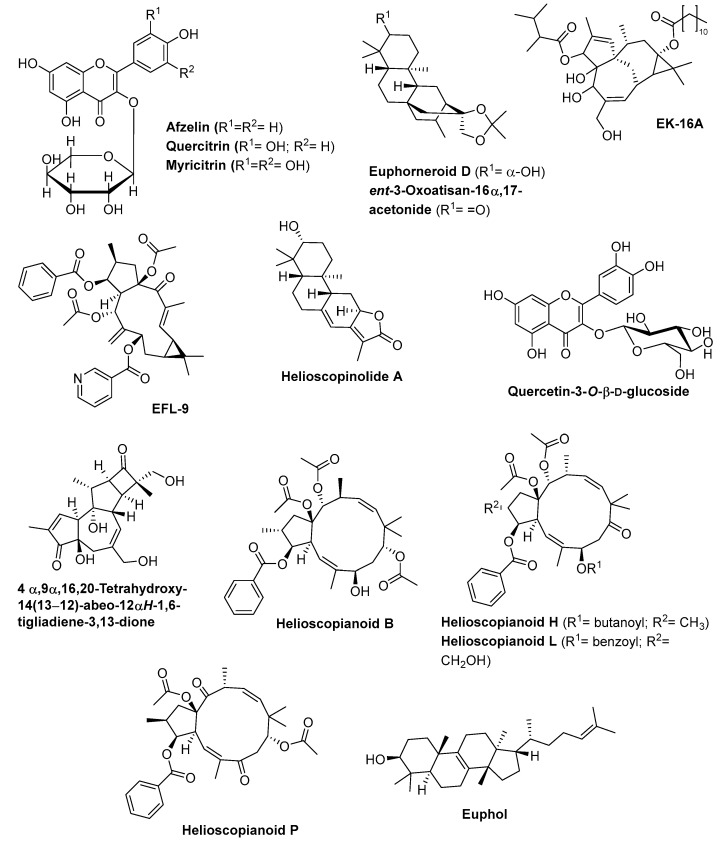
Chemical structures of in vitro bioactive *Euphorbia* compounds.

Methanolic extract of *E. hirta* aerial parts exhibited 90% growth inhibition against *Plasmodium falciparum* at 5 µg/mL and low toxicity against KB 3-1 cells, demonstrating its potential as antimalarial agent [96]. From this extract and by a bio-guided methodology, the authors isolated three flavonol glycosides—afzelin, quercitrin, and myricitrin (Figure 2)—that exhibit the ability to significantly inhibit the proliferation of the protozoan responsible for malaria disease *Plasmodium falciparum* strains FCR-3 (cycloguanil-resistant from Gambia) and CDC1 (chloroquine sensitive), with similar IC_50_ values 2.5 to 11.6 µM [96]. Quercitrin is able to arrest irreversibly the life cycle of the parasite at the trophozoite stage [96].

Several diterpenes isolated from stem bark of *E. neriifolia* exhibit anti-HIV-I activity [97]. Among these diterpenes, euphorneroid D and *ent*-3-oxoatisan-16α,17-acetonide (Figure 2) were the most active compounds, with EC_50_ values of 34 µM and 24 µM respectively, although much less active than the reference compound (zidovudine, EC_50_ = 0.0019 µM). Besides these diterpenes, the ingenol derivative EK-16A (Figure 2), isolated from the *E. kansui* S.L.Liou ex S.B.Ho, can also be useful as antiviral agent against HIV-1 [98]. In fact, this compound is a PKCγ activator, 200-fold more potent (IC_50_ = 3.53 to 4.06 nM) than prostratin (IC_50_ = 768 to 865 nM) in reactivating latent HIV-1 without exerting detectable cytotoxicity on C11 and the J-Lat 10.6 cells viability and T cell activation. In addition, this compound exhibits a synergistic effect with other activators of HIV-1 from latency like prostratin, 5-azacytidine, and romidepsin [98].

The seeds of *E. lathyris* contain many natural macrocyclic diterpenes with a lathyrane skeleton [46,99], five of which were evaluated against A549, MDA-MB-231, MCF-7, KB, and KB-VIN cancer cell lines [99]. The compound EFL-9 (Figure 2) exhibits the strongest cytotoxicity against all cell lines (IC_50_ values ranging from 5.7 to 8.4 µM against four of the five cell lines tested). The most important is its similar toxicity against the multidrug-resistant cancer cell line KB-VIN and the parental cell KB line (IC_50_ values of 5.7 and 6.1 µM, respectively), while the anticancer taxol exhibits a highly differentiated toxicity to these cell lines (IC_50_ values of 1.9 µM and 6.7 nM, respectively) [99]. The nicotinate ester at C-7 on the EFL-9 compound seems to play a crucial role in the cytotoxicity against the KB-VIN cell line. Additionally, the cytotoxicity of EFL-9 is due to its action on actin filament aggregation as well as on partial disruption of microtubules networks [99].

Helioscopinolide A (Figure 2), one jolkinolide-type diterpenoid isolated from the ethanolic extract of *E. helioscopia* whole plant exhibits a strong cytotoxic activity against HeLa cells line (IC_50_ = 0.11 µM), being even more active than the adriamycin used as positive control (IC_50_ = 0.41 µM) [100]. Helioscopinolide A exhibits moderate activity against MDA-MB-231 cells line (IC_50_ = 2.1 µM) and is much lesser active than Adriamycin (IC_50_ = 0.34 µM) [100].

According Safwat et al. [101], quercetin-3-*O*-β-d-glucoside (Figure 2), a safe compound isolated from 70% methanol extract of *E. paralias* whole plant, in addition to exhibiting moderate toxicity against HepG2 and A549 cancer cell lines (IC_50_ values of 41 and 36 µM, respectively), exhibits the ability to inhibit the glutamine synthetase enzyme (IC_50_ = 0.048 µM). Taking into account that this enzyme is identified as a potential target in the development of new antimycobacterial agents, once it plays a significant role as virulence factor of *Mycobacterium tuberculosis*, Safwat et al. [101] showed that quercetin-3-*O*-β-d-glucoside could be used as an antituberculotic agent.

Several isolated diterpenoids from the roots of *E. ebracteolata* Hayata were reported to inhibit human carboxylesterase 2 (hCE-2), a human enzyme able to metabolize clinical drugs, resulting in adverse clinical reactions such as the reduction of biological availability of the drugs [102]. The compound 4αβ,9α,16,20-tetrahydroxy-14(13→12)-abeo-12α*H*-1,6-tigliadiene-3,13-dione (Figure 2) exhibits the strongest competitive inhibitory activity (IC_50_ = 3.88 µM, with Ki = 4.94 µM), a higher activity than the hCE-2 inhibitor BNNP (IC_50_ = 5.60 µM). On the other hand, this compound has non-toxic effect against HCT-116, HepG2, BGC-823, H460, and SK-OV-3 cell lines (IC_50_ >100 µM) [102], which is an advantage in its application as an hCE-2 inhibitor.

Helioscopianoid P and helioscopianoid H (Figure 2) are two jatrophane-type diterpenoids isolated from the whole plants of *E. helioscopia*, at 20 µM, exhibit inhibitory effects on P-glycoprotein in adriamycin (ADM)-resistant human breast adenocarcinoma cell line (MCF-7/ADR), while helioscopianoid L, helioscopianoid H and helioscopianoid B (Figure 2), also jatrophane-type diterpenoids isolated from the same species, exhibit neuroprotective effects against serum deprivation-induced PC12 cell damage and rotenone-induced PC12 cell damage [103].

The main component of *E. umbellata* and *E. tirucalli* latex is the tetracyclic triterpene alcohol euphol (Figure 2) [104,105]. This compound exhibits cytotoxic effect against several leukemia, colorectal, melanoma and glioma cancer cell lines [104,105]. When tested against Jurkat, HL-60, K-562 B16F10 and HRT-18 cell lines, euphol is toxic mainly against K-562 cell line (34.44 µM) by apoptosis induction, with non-toxicity to blood cells (SI > 2), but low selectivity to 3T3 cell line (SI = 0.55) [104]. On the other hand, euphol is particularly active against glioma cells (including primary, paediatric and adult glioma cell lines), with IC_50_ values range from 5.98 to 31.05 µM, much more active than the temozolomide, a clinical drug used to treat some brain cancers (IC_50_ values range from 97.00 µM to 1 mM) and with higher selective cytotoxicity index (0.64–3.36) than temozolomide (0.11–1.13) [105].

### 5.2. In Vivo Studies

The first significant in vivo studies reported on *Euphorbia* extracts or pure compounds were published in the 1990s. For example, the lyophilized *E. hirta* decoction exhibits antidiarrheal activity on diarrhoea induced by castor oil, arachidonic acid, and prostaglandin E2, but is inactive when the diarrhoea is induced by magnesium sulphate [106]. The flavonoid quercitrin (Figure 2) isolated from the previously reported lyophilized *E. hirta* decoction, exhibits antidiarrheal activity (at doses of 50 mg/kg), in castor oil- and PGE2-induced diarrhoea in mice [107], although the same authors showed that quercitrin activity is due to the glycone quercetin released in the intestine [107]. On the other hand, the water extract of *E. hirta* leaves, in adult Wister rats at 50–100 mg/kg dose, exhibits diuretic effect and increase the excretion of Na^+^, K^+^ and HCO_3_ [108].

However, the highest incidence of in vivo studies involving *Euphorbia* species has occurred in the last few years.

The 80% hydroethanolic extract of *E. tirucalli* latex at 0.250 mg (daily for 21 and 35 days) seems to be able to induce significant increases in T_H_1 cytokines (GM-CSF, IL-2), T_H_2 cytokines (IL-6), and chemokines (IL-1β, RANTES) and thus incite immunological stimulation and improve the immune system on adult male Sprague Dawley rats [109].

A steroid and terpenoid-rich fractions were isolated from the hydroethanolic extract of *E. tirucalli* root that, at 60 mg/kg, exhibits better protection against peripheral nociceptive pain on acetic acid induced abdominal constrictions mice model than the analgesic aceclofenac sodium and better anti-inflammatory activity in carrageenan-induced mice model than indomethacin [110].

On the other hand, the 70% hydroethanolic extract of *E. supina* Raf. (this name is a synonym of *E. maculata* L.), at 10 mg/mL, is able to significantly reduce the ear thickness and number of inflammatory cells on the *Propionibacterium* acnes-induced skin inflammation by inhibition of pro-inflammatory cytokines expression and the MAPK signalling pathway [111].

The wound-healing activity of different extracts (*n*-hexane, ethyl acetate, and methanol) from aerial parts of different *Euphorbia* species (*E*. *helioscopia*, *E. characias* subsp. *Wulfenii* (Hoppe ex W.D.J.Koch) Radcl.-Sm., *E. macroclada* Boiss., *E. seguieriana* subsp. *seguieriana* (this name is a synonym of *E. seguieriana* Neck.), and *E. virgata* Waldst. & Kit. (this name is a synonym of *E. esula* subsp. *tommasiniana* (Bertol.) Kuzmanov), was evaluated in vivo by Özbilgin et al. [112]. The authors showed that the methanol extract of *E. characias* subsp. *wulfenii* was the most active, significantly reducing the linear incision wound (43.04%) while the circular excision wound decreased 65.24%, with a significant decrease in wound inflammation (34.74%) in relation to the control groups. These activities were further linked with the quercetin glycosides identified in the extract [113].

A comparative study with several species, including *E. helioscopia*, *E. lactea* Haw., and *E. nivulia* Buch.-Ham. showed that the ethanol extract from the latter (at 100 mg/kg) was the one that caused the greatest anticonvulsant effects and highest reduction in the progression of epileptogenesis in pentylenetetrazole-induced kindling model of epilepsy in mice [114].

It was well demonstrated that the lyophilized decoction of fresh whole *E. hirta* had great potential against dengue virus since, after 14 days of administration at 100 mg/kg dose, it significantly increased platelet counts in rats, also causing a decrease in coagulation time and bleeding [115].

As shown above and in a recent review on the subject [116], several reports have suggested that *Euphorbia* plant possesses, in in vitro assays, considerable cytotoxic potential. Some in vivo studies also corroborate the anticancer potential and safety of *Euphorbia* extracts and compounds. For example, the methanolic extract of *E. triaculeata* Forssk., when tested in albino mice at 250 to 1000mg/kg body weight, is not genotoxic or clastogenic, while the pre-treatment with this extract inhibits the clastogenicity induced by cyclophosphamide without impairing its cytotoxic potential [117].

The tetracyclic triterpene alcohol, euphol (Figure 2), showed antitumoural effects in GAMG glioma model through the activation of autophagy-associated cell death [105].

**Figure 3:**
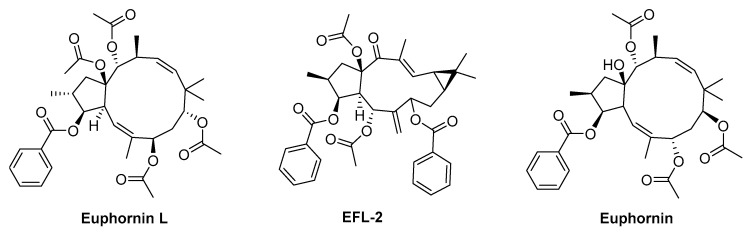
Chemical structures of some in vivo bioactive *Euphorbia* compounds.

Euphornin L (Figure 3), a relatively abundant jatrophane diterpenoid from *E. helioscopia*, exhibits outstanding lipid-lowering effect on golden Syrian hamsters fed with a high-fat diet at a well-tolerated dose of 30mg/kg (p.o.) [118]. In fact, the authors observed a significant decrease on CHOL and LDL-C levels in the treatment group when compared with control group, while other parameters (HDLC, TG, and body weight) did not suffer significant changes [119].

Euphorbia factor L2 (EFL-2, Figure 3), a secondary metabolite isolated from seeds of *E. lathyris*, was tested to treat lipopolysaccharide (LPS) induced acute lung injury (ALI) in mice [119]. EFL-2 at concentration of 40 mg/kg causes an attenuation of the pathological changes in the lung by significant suppression of the recruitment and transmigration of inflammatory cells, specifically neutrophils [119]. The same compound was tested, using SMMC7721 xenograft BALB/c nude mice, to evaluate its effect on human hepatocellular carcinoma [120]. EFL-2, administered at 25 and 50 mg/kg for 14 days, is able to inhibit the growth of hepatocellular carcinoma in BALB/c athymic nude mice, causing a decrease in tumour volume and weight, mainly by targeting STAT3 and AKT inactivation during TGF-β-induced EMT and metastasis [120].

From *E. helioscopia* aerial parts were isolated several secondary metabolites that exhibit vasodepressive activity, being the euphornin (Figure 3) the most active, causing a significant reduction in direct blood pressure (42 mmHg) in an adult male Wistar albino rat model during 70 min [121].

### 5.3. Clinical Studies

There are very few clinical studies involving *Euphorbia* species. In a clinical trial whose results were published in 2011 [122], skin cancer lesions (basal cell carcinomas, squamous cell carcinomas, and intraepidermal carcinomas) were treated topically with 100–300 µL of *E. peplus* latex (daily for three days). The results showed this latex exhibits a clinical response that is comparable to existing non-surgical treatments used in clinical therapeutics against human non-melanoma skin cancers. The active ingredient of *E. peplus* latex has been identified as ingenol mebutate, the active principle of the clinical medicine Picato® used in the treatment of actinic keratosis [24].

The *E. hirta* extract is able to cause an increase in the number of platelets in dengue patients from 30–55 age group and a decrease in flu-like symptoms in 70% of the patients. However, no identical effect was observed in the 14–25-age group [123].

A clinical study on phase 2 to determine the safe and efficacious dose of *E. prostrata* for control of per rectal bleeding in patients with first- and second-degree haemorrhoids is now complete (Clinical Trials Identifier: NCT01041911) [124]. In that trial, 82% of the patients had complete cessation of bleeding at the end of two weeks and thus demonstrate that *E. prostrata* can be used as an effective therapeutic agent and without adverse effects in the treatment of early stage haemorrhoids [125].

A clinical trial involving *E. kansui* (an *E. kansui* extract powder prepared as tea) is currently in the recruitment phase and intends to evaluate the immune response of HIV+ individuals to this herbal supplement and its dependence with the dose applied. Also, the safety of the herbal preparation is evaluated (Clinical Trial Identifier: NCT02531295) [124].

## 6. Conclusions

The *Euphorbia* species are plants well known for their applications, especially its latex, in traditional medicine around the world. Their chemical composition may vary according to the species, to the part of the plant, and the applied extraction methodology. This variability could also be influenced by the different habitats, seasons, and dates of collection. Quantitative and qualitative analysis have been developed for the compounds’ identification, being the major constituents of essential oils oxygenated sesquiterpenes followed by sesquiterpene hydrocarbons.

Additionally, several studies reported and discussed above, confirmed, in vitro, in vivo, and in clinical trials, the biological activities of *Euphorbia* extracts and pure compounds. These compounds and extracts could be applied to the treatment of different diseases mainly related to microbial infections, as well as inflammation and cancer.

*Euphorbia* plants have great potential as a source of bioactive extracts and pure compounds, which may lead to the development of new drugs for clinical use.

## Figures and Tables

**Table 1:** Chemical composition and biological activities of *Euphorbia* essential oils.

Species	Origin	Raw Material	Extraction Method	Main Components ^a^ (%)	Most Relevant Biological Activities	Ref.
*E. acanthothamnos* Heldr. & Sart. ex Boiss.	Greece	Inflorescences	Steam distillation	Phytol (28.3), phytol acetate (9.3), β-caryophyllene (7.5)	not evaluated	[59]
*E. apios* L.	Greece	Inflorescences	Steam distillation	Germacrene D (30.0), heptacosane (12.7), β-caryophyllene (10.0), tricosane (6.5), pentacosane (6.0)	not evaluated	[59]
*E. characias* L.	Greece	Inflorescences	Steam distillation	Nonanal (22.8), phytol (13.5), pentacosane (8.5), heptacosane (7.4), palmitic acid (5.7), nonacosane (5.6)	not evaluated	[59]
*E. cotinifolia* L. (syn. *E. caracasana* (Klotzsch & Garcke) Boiss.)	Venezuela	Leaves	Hydro-distillation	β-Caryophyllene (39.3), germacrene-D (21.5%), α-copaene (9.3), α-humulene (5.2)	not evaluated	[60]
*E. dendroides* L.	Greece	Inflorescences	Steam distillation	Heptacosane (10.5), pentacosane (6.0), 4-terpineol (5.5), tricosane (5.0)	not evaluated	[59]
*E. densa* Schrenk	Syria	Aerial parts	Hydro-distillation	1,8-Cineole (18.87), linalool (13.61), carvacrol (13.32), (*E*)-caryophyllene (10.29)	Radical scavenging activity (EC_50_ = 0.35 µg/mL) lower than BHA (EC_50_ = 0.135 µg/mL)	[61]
*E. fischeriana* Steud.	China	Roots	Steam distillation	Eudesmol (18.22), *p*-menth-8-en-2-ol (9.36), caryophyllene oxide (8.61), selinenol (6.83)	Radical scavenging activity (IC_50_ = 57.2 µg/mL) similar to ascorbic acid (IC_50_ = 63.1 µg/mL) but lower than BHT (IC_50_ = 26.1 µg/mL)	[62]
*E. fragifera* Jan	Italy	Inflorescences	Steam distillation	Carvacrol (61.55), carvon (9.22), β-caryophyllene (5.80)/geraniol (59.65), β-caryophyllene (9.05)	not evaluated	[63]
*E. gaillardotii* Boiss. & Blanche	Turkey	Aerial parts	Hydro-distillation	Arachidic acid (32), hexatriacontane (8.7), mint furanone (8.4), palmitic acid (8.0), tetratetracontane (6.2), octadecane (5.6), α-silenene (5.2)	Anti-lipid peroxidation activity (IC_50_ = 14.8 µg/mL) similar to α-tocopherol, but much lower radical scavenging activity than BHT.	[64]
*E. golondrina* L.C.Wheeler	Cameroon	Leaves	Steam distillation	Caryophyllene oxide (14.16), 2-pentadecanone (13.78), camphor (9.41), phytol (5.75)	not evaluated	[65]
*E. hebecarpa* Boiss.	Iran	Aerial parts	Hydro-distillation	α-Bisabolol (31.2), *cis*-cadin-4-en-7-ol (20.1), *trans*-piperitol (8.6), cis-*p*-menth-2-en-1-ol (6.4), *trans*-*p*-menth-2-en-1-ol (6.2)	not evaluated	[66]
*E. helioscopia* L.	Greece	Inflorescences	Steam distillation	Phytol (21.2), β-caryophyllene (10.0), behenic acid methyl ester (8.1), myristic acid methyl ester (5.5)	not evaluated	[59]
*E. helioscopia* L.	Turkey	Aerial parts	Hydro-distillation	β-Cubebene (19.3), palmitic acid (12.2), caryophyllene oxide (11.7), τ-elemene (9.3), spathulenol (9.3), phytol (6.9), hexahydrofarnesly acetone (5.3)	Low antioxidant and antiacetylcholinesterase activity, moderate butyrylcholinesterase and similar anti-urease activity to thiourea.	[67]
*E. heterophylla* L.	Nigeria	Leaves	Hydro-distillation	3,7,12,15-Tetramethyl-2-hexadecen-1-ol (12.30), stearic acid (11.21), oleic acid (10.42), linoleic acid (8.97), 1,2-epoxy-cyclododecane (7.91), 13-tetradece-11-yn-1-ol (7.83), 7,10-hexadecadienal (7.62), 1,2,15,16-diepoxyhexadecane (6.37), phytol (6.32), 2-monopalmitin (5.43)	Toxic to brine shrimp larvae (LC_50_ = 21.7 µg/mL). Radical scavenging activity similar to ascorbic acid, lower than BHA but higher than α-tocopherol at 250 µg/mL.	[68]
*E. heterophylla* L.	Nigeria	Stems	Hydro-distillation	Stearic acid (11.21), oleic acid (10.42), linoleic acid (8.97), 1,2-epoxy-cyclododecane (7.91), 13-tetradece-11-yn-1-ol (7.83), 7,10-hexadecadienal (7.62), 1,2,15,16-diepoxyhexadecane (6.37), phytol (6.32), 2-monopalmitin (5.43), 2-aminoethoxyethynediyl methyl ester (5.40)	Very toxic to brine shrimp larvae (LC_50_ = 8.94 µg/mL). Radical scavenging activity similar to ascorbic acid, lower than BHA but higher than α-tocopherol at 250 µg/mL.	[68]
*E. heterophylla* L.	Egypt	Aerial parts	Hydro-distillation	1,8-Cineole (32.0), camphor (16.5), β-elemene (5.9 )	Radical scavenging activity (IC_50_ 325.3 µL/L) lower than ascorbic acid (204.4 µL/L).	[69]
*E. hirta* L.	Lagos	Leaves	Hydro-distillation	Phytol and its isomeric forms (34.8), 6,10,14-trimethyl-2-pentadecanone (12.37), hexadecanal (7.63), palmitic acid (6.26)	not evaluated	[70]
*E. macroclada* Boiss.	Turkey	Aerial parts	Hydro-distillation	Tetratetracontane (42.7), hexatriacontane (12), mint furanone (6.0)	Anti-lipid peroxidation activity (IC_50_ = 14.8 µg/mL) similar to α-tocopherol. Lower radical scavenging activity than BHT but higher than *E. gaillardotii* essential oil.	[64]
*E. macrorrhiza* C.A.Mey. ex Ledeb.	China	Aerial parts	Hydro-distillation	Acorenone B (16.72), (+)-cycloisosativene (14.94), 3β-hydroxy-5α-androstane (10.62), β-cedrene (8.40), copaene (7.37), palmitic acid (5.68)	Cytotoxic activity against Caco-2 cell line (IC_50_ = 78.32 µg/mL), antibacterial activity against *Staphyloccocus aureus* (MIC = 5.6 µg/mL) but lower than ampicillin (MIC = 0.25 µg/mL)	[71]
*E. macrorrhiza* C.A.Mey. ex Ledeb.	China	Roots	Hydro-distillation	Acorenone B (25.80), (+)-cycloisosativene (12.40), β-cedrene (7.98), copaene (6.29), 3β-hydroxy-5α-androstane (5.52)	Cytotoxic activity against Caco-2 cell line (IC_50_ = 11.86 µg/mL), antibacterial activity against *Staphyloccocus aureus* (MIC = 2.8 µg/mL) but lower than ampicillin (MIC = 0.25 µg/mL)	[71]
*E. pekinensis* Rupr.	China	Roots	Steam distillation	Agarospirol (49.23), hedycargol (20.66)	not evaluated	[72]
*E. pilosa* L.	India	Aerial parts	Hydro-distillation	Phytol (5.75), *n*-pentadecanal (5.12)	not evaluated	[73]
*E. rigida* M.Bieb.	Greece	Inflorescences	Steam distillation	Heneicosane (13.8), heptacosane (12.7), β-caryophyllene (9.4), linalool (6.7), pentacosane (6.5)	not evaluated	[59]
*E. sanctae-caterinae* Fayed	Egypt	Aerial parts	Hydro-distillation	Valencene (16.01), (+) spathulenol (15.41), (-)-caryophyllene oxide (10.50), limonene (7.66)	not evaluated	[74]
*E. sanctae-caterinae* Fayed	Egypt	Aerial parts	Microwave-assisted	Butyl hydroxyl toluene (25.58), β-eudesmol (13.67), 6-*epi*-shyobunol (11.83), (+) spathulenol (10.32), thymol (7.00)	not evaluated	[74]
*E. teheranica* Boiss.	Iran	Aerial parts	Hydro-distillation	Elemol (57.5), β-caryophyllene (8.1%), caryophyllene oxide (7.8%)	not evaluated	[75]
*E. thymifolia* L.	India	Aerial parts	Steam distillation	Palmitic acid (33.03), phytol (10.367), myristic acid (6.58)	not evaluated	[76]
*E. tithymaloides* L.	Bangladesh	Aerial parts	Steam distillation	Eugenol (22.52), phenyl ethyl alcohol (14.63), 3-pentanol (9.22), caryophyllene oxide (7.73), isoeugenol (7.32), pentadecanol (5.14), spathulenol (5.11)	Radical scavenging activity (DPPH IC_50_ = 13.67 µg/mL) higher than BHA (IC_50_ = 18.26 µg/mL).	[77]

^a^ Compounds with content higher than 5%.

**Table 2:** Antibacterial and antifungal activity of *Euphorbia* extracts.

*Euphorbia* Species	Tested Extract	Activity against	Highest Level of Activity *	Ref.
*E. royleana* Boiss.	Water Methanol Hexane	*Escherichia coli*, *Bacillus subtilis*, *Pasteurella multocida*, *Aspergillus niger*, *Fusarium solani*	Hexane extract against *A. niger* 14.00 ± 1.00 mm (terbinafine: 25.66 ± 1.69 mm)	[84]
*E. hirta* L. *E. tirucalli* L. *E. neriifolia* L.	Methanol	*Pseudomonas aeruginosa*, *Staphylococcus aureus*, *Bacillus subtilis*, *Escherichia coli*, *Cryptococcus luteolus*, *Candida albicans*, *Candida tropicalis*, *Candida neoformans*	Extract of *E. tirucalli* against *P. aeruginosa* 14.00 ± 0.00 mm.	[85]
*E. neriifolia* L.	Chloroform Ethanol Ethyl acetate Butanol Water	*Staphylococcus aureus*, *Klebsiella pneumonia*, *Escherichia coli*, *Proteus vulgaris*, *Pseudomonas fluorescents*	Chloroform extract against *P. vulgaris* 8 ± 0.4 mm.	[86]
*E. paralias* L.	Acetone Chloroform	*Salmonella enterica*, *Escherichia coli*, *Bacillus subtilis*, *Pseudomonas aeruginosa*, *Staphylococcus aureus*, *Candida albicans*	Chloroform extract against *B. subtilis* MIC = MBC = 15 µg/mL (Gentamicin, MBC = 15 µg/mL)	[87]
*E. granulata* Forssk. *E. helioscopia* L. *E hirta* L.	Ethanol	*Klebsiella pneumonia*, *Proteus vulgaris*, *Streptococcus pyogenes*, *Escherichia coli*, *Staphylococcus aureus*, *Staphylococcus epidermidis*, *Geotrichum candidum*, *Microsporum canis*, *Trichophyton mentagrophytes*, *Aspergillus fumigatus*, *Candida albicans*, *Candida tropicalis*	Extract of *E. hirta* against *S. aureus* and *M. canis* MIC 1.95 µg/mL (ampicillin MIC = 0.06 µg/mL; amphotericin B MIC = 1.95 µg/mL)	[88]
*E. hirta* L.	Ethanol	*Escherichia coli*, *Staphylococcus aureus*, *Pseudomonas aeruginosa*, *Bacillus subtilis*	Against *S. aureus* MIC = 22.55 mg/mL	[89]
*E. hirta* L.	Hexane Dichloromethane Ethyl acetate Ethanol	*Enterobacter aerogenes*, *Escherichia coli*, *Klebsiella pneumonia*, *Proteus mirabilis*, *Proteus vulgaris*, *Pseudomonas aeruginosa*, *Salmonella typhi*, *Shigella dysenteriae*, *Staphylococcus aureus*, *Bacillus subtilis*	Ethanol extract against *S. typhi* MIC= 31 µg/mL (chloramphenicol MIC = 62 µg/mL)	[90]
*E. characias* L.	Ethanol Water	*Staphylococcus aureus*, *Bacillus cereus*, *Listeria monocytogenes*, *Escherichia coli*, *Salmonella typhimurium*, *Candida albicans*, *Saccharomyces cerevisiae*, *Aspergillus flavus*, *Penicillium chrysogenum*	Ethanol extract against *B. cereus* MIC = 312.5 µg/mL (ampicillin MIC = 10 µg/mL)	[91]

* Express as diameter of inhibition zone (mm), as minimum inhibitory concentration, MIC or as minimum bactericidal concentration (MBC) (µg/mL). When available, the antimicrobial activity of the reference compound is presented.
